# Which Mosquito Is the Enemy?

**Published:** 1899-07-08

**Authors:** 


### WHICH MOSQUITO IS THE ENEMY?

In his inaugural lecture on tropical medicine at University College, Liverpool, Major Ronald Ross described the position in which our knowledge of the malaria parasite at present stands, and more especially pointed out how far we are likely to be able to control the disease by attacking the particular mosquito which acts as the definitive host of the organism. The latter is an interesting point and one worthy of the most careful attention. Recognising, as is now admitted on all hands, that the malarial parasite is carried to man by the bite of the mosquito in whose body it attains maturity, the question is, what mosquito? for on the answer to this depends our mode of attack on the disease. It appears that there are many kinds of mosquitos which differ not only in form and colour, but in their habits and in the localities which they select for breeding purposes; and as it is only when the mosquito is in the larval stage that we can attack it with any hope of success the question of the breeding place is the key to the position. According to Major Ross there are, at any rate in India, two main classes of mosquito, one of which, that of the genus Culex, specially infests towns and houses, breeding in pots and tubs of water, in cisterns, drains, wells, and artificial water reservoirs, and may thus be called the domestic mosquito; and the other, of the genus Anopheles, which may be called the rural mosquito, which does not breed in vessels and artificial collections of water, but in natural ponds and rain-water puddles. It is in various species of this latter class of mosquitos that the malaria parasite has been artificially cultivated, and Major Ross hopes that it is among them that the natural host of the parasite will be found. It has to be noted that the sort of pond or puddle suitable for the breeding of these mosquitos is somewhat peculiar, and that, in consequence, their breeding places are probably not very numerous. Thus to breed these mosquitos the puddles must be large enough to be permanent for a few days to allow the larvæ to mature; they must not be so large as to contain minnows, which devour the larvæ with avidity; and they must not be liable to be scoured out by every shower of rain. Hence the places in which these mosquitos can breed freely are comparatively few in number, and might possibly be stopped up or otherwise dealt with so as to exterminate this particular sort of mosquito in malarious districts. If then, as Major Ross opines, it is by mosquitos of this class that the parasite is conveyed to man, such proceedings might be fairly hoped to have the effect of extirpating malaria from the districts in question. This, then, is the next question, and is one which demands a definite answer. It is no use wasting our energies in measures for the suppression of the common mosquito, whose opportunities for breeding are so numerous that its suppression is practically hopeless, if it is the other and much more easily got at species which does the mischief.

